# Self-Oscillating Liquid Crystal Elastomer Helical Spring Oscillator with Combined Tension and Torsion

**DOI:** 10.3390/polym15153294

**Published:** 2023-08-03

**Authors:** Dali Ge, Yuntong Dai, Kai Li

**Affiliations:** 1 School of Civil Engineering, Anhui Jianzhu University, Hefei 230601, China; dalige@ahjzu.edu.cn (D.G.); daiytmechanics@ahjzu.edu.cn (Y.D.); 2 Institute of Advanced Technology, University of Science and Technology of China, Hefei 230001, China

**Keywords:** helical spring, self-oscillation, liquid crystal elastomers, optically responsive, tension and torsion

## Abstract

Self-oscillation is the autonomous maintenance of continuous periodic motion through energy absorption from non-periodic external stimuli, making it particularly attractive for fabricating soft robots, energy-absorbing devices, mass transport devices, and so on. Inspired by the self-oscillating system that presents high degrees of freedom and diverse complex oscillatory motions, we created a self-oscillating helical spring oscillator with combined tension and torsion under steady illumination, among which a mass block and a liquid crystal elastomer (LCE) helical spring made with LCE wire are included. Considering the well-established helical spring model and the dynamic LCE model, a nonlinear dynamic model of the LCE helical spring oscillator under steady illumination is proposed. From numerical calculation, the helical spring oscillator upon exposure to steady illumination possesses two motion regimes, which are the static regime and the self-tension–torsion regime. Contraction of the LCE wire under illumination is necessary to generate the self-tension–torsion of the helical spring oscillator, with its continuous periodic motion being maintained by the mutual balance between light energy input and damping dissipation. Additionally, the critical conditions for triggering the self-tension–torsion, as well as the vital system parameters affecting its frequencies and amplitudes of the translation and the rotation, were investigated in detail. This self-tension–torsion helical spring oscillator is unique in its customizable mechanical properties via its structural design, small material strain but large structural displacement, and ease of manufacture. We envision a future of novel designs for soft robotics, energy harvesters, active machinery, and so on.

## 1. Introduction

Self-oscillation is the autonomous maintenance of continuous periodic motion through periodically absorbing energy from non-periodic external stimuli [1,2]. Self-oscillatory phenomena are abundant in nature, such as leaves flutter, neural impulses, heartbeats, and circadian clocks. Generally, the motion frequency is determined by its own characteristics, and thus it features good robustness [3,4]. Additionally, it has no requirements of an additional complex controller and a portable battery [5]. In view of these preponderances, self-oscillation systems hold great promise for applications in autonomous robotics [6,7,8,9], energy-absorbing devices [10,11], self-propelling devices [12], mass transport devices [13,14], and so on.

In recent years, various stimuli-responsive materials have been used to synthesize self-oscillating systems, e.g., dielectric elastomers [15], hydrogels [16,17], ionic gels [18], thermally responsive polymer materials [19], and liquid crystal elastomers (LCEs) [7,20], etc. Furthermore, a vast number of self-sustained motion modes have been constructed, such as bending [21,22,23,24,25], buckling [26,27,28,29], eversion or inversion [30,31], floating [32,33], jumping [34,35,36], rolling [8,37,38,39], curling [40], rotation [41,42], spinning [43], swimming [9,44], swinging [45,46], stretching and shrinking [21,47,48], snap-through [49,50], torsion [21,51], vibration [21,52,53], and even synchronized motion of several coupled self-oscillators [54,55]. These self-sustained motions often originate from nonlinear feedback mechanisms, including self-shadowing [27,31], coupling large deformation with chemical reaction [20], photothermal solvent evaporation [56], and photothermal surface tension gradients [57].

Among the various effective stimuli, optical excitation is the most favorable stimulus which can be precisely controlled, non-contact driven, programmable, and eco-efficient [21,42]. LCE is a vital and particularly promising optically responsive material, which is synthesized by combining anisotropic rod-like liquid crystal molecules with stretchable long-chain polymers [58]. When exposed to environmental stimuli such as light [21], heat [25], electricity [59], and magnetism [60], the rotation or phase transition of the liquid crystal monomer molecules induces a configurational change, resulting in macroscopic deformation [61]. Typically, the optically responsive LCE exhibits the behaviors of fast response, large intrinsic deformation, as well as reversible deformation. These special attributes facilitate the realization of light-driven self-oscillation in diverse fashions [34,40], which leads to great progress in creating LCE-based light-fueled self-oscillation systems [34,35,61,62].

**Figure 1:**
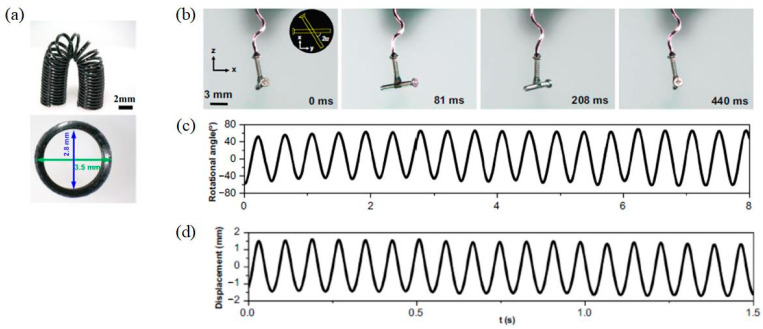
Light-driven self-oscillation of LCE helical spring system [51]: (**a**) LCE helical spring, (**b**) experimental snapshots of self-oscillation, (**c**) time history of self-oscillation in rotational direction, (**d**) time history of self-oscillation in translational direction.

The more types of oscillation modes are available, the more sophisticated autonomous devices may potentially be constructed. Most of the light-driven self-oscillation systems reported to date are based on simple bending deformation [37,61] or tension/compression deformation [47,48,49] as the main degree of freedom of motion. Recently, the self-oscillation of an LCE helical spring system under steady illumination was reported. In the experiment [51], this self-oscillating system presented high degrees of freedom and diverse complex oscillatory motions, as shown in Figure 1. Inspired by this research, we creatively constructed a helical spring oscillator consisting of a mass block and an LCE helical spring made of LCE wire, which can self-oscillate with combined tension and torsion under steady illumination. In contrast to previous self-torsion systems in which the self-torsion originates from laser irradiation on the edge of the cantilever [21] or asymmetric distribution of strain/stress over the cross-section [22], the self-torsion of the LCE helical spring oscillator in our research results from a three-dimensional helix structure made by a simple one-dimensional wire. It features unique advantages of customizable mechanical properties through structural design, small material strain but large structural displacement, and ease of manufacture [63,64,65,66,67]. This created self-tension–torsion system based on an LCE helical spring may constitute novel designs for soft robotics, energy harvesters, active machinery, and so on.

The remainder of this study is organized as follows. In Section 2, we first propose a nonlinear dynamic model of the self-oscillating LCE helical spring oscillator with combined tension and torsion under steady illumination, in which the well-established helical spring model and dynamic LCE model are employed. Then, we derive the corresponding governing equations. Following that, two motion regimes (static regime and self-tension–torsion regime) of the helical spring oscillator under steady illumination are outlined, and the detailed discussion about the mechanism of self-tension–torsion is carried out in Section 3. Subsequently, Section 4 investigates the critical conditions triggering self-tension–torsion, and the system parameters affecting its frequency and amplitude. Finally, we conclude our research in Section 5.

## 2. Model and Formulation

In this section, a theoretical model is proposed for the LCE helical spring oscillator under steady illumination. The main contents include the dynamics of the helical spring oscillator, the helical spring model, and the evolution of the cis-isomers number fraction in the LCE wire, its nondimensionalization, and the solution method of the differential governing equations with variable coefficients.

### 2.1. Dynamics of the Helical Spring Oscillator

**Figure 2:**
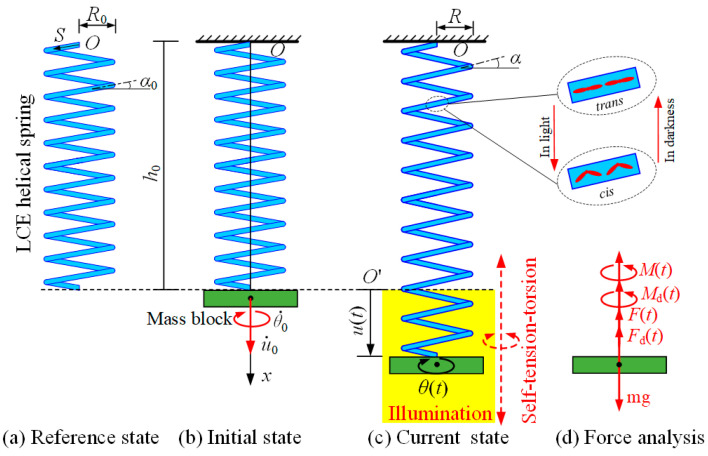
Schematics of a self-tension–torsion helical spring oscillator: (**a**) reference state, (**b**) initial state, and (**c**) current state. (**d**) The force analysis diagram of mass block. Under the steady illumination, the helical spring oscillator can maintain a continuous periodic self-tension–torsion.

Figure 2 shows an LCE helical spring oscillator under steady illumination, which possesses a mass block and an optically responsive LCE helical spring made of LCE wire with negligible mass. The helical spring is fixed at point *O*, and its other end is hung with a mass block of mass m and inertia moment J. In the reference state, the helical spring is stress-free, with helix angle $\alpha_{0}$, height $h_{0}$ and coil radius $R_{0}$, as shown in Figure 2a. Therefore, the original length of the LCE wire can be easily calculated as $s_{0}=h_{0}/\sin\alpha_{0}$. The azobenzene molecules in the nematic LCE wire are oriented along the length direction. It is well-known that illumination will induce the LCE wire to undergo reversible contraction, and this light-driven contraction will recover in the dark [11,48,58].

The illuminated zone is denoted by the yellow shaded area, and the rest is in the dark zone, as shown in Figure 2b. In the initial state, the mass block is released where the helical spring is stress-free, and it will move downward due to gravity. Herein, the helical spring is stretched and subjected to an axial spring force F(t) at time t, that leads its height to increase and results in part of the LCE wire moving into the illumination zone, as shown in Figure 2c. Since the azobenzene liquid crystal molecules transform from the straight *trans* state to the bent *cis* state in illumination (shown in Figure 2c), the part of the LCE wire in the illumination zone contracts in the length direction and the length of the LCE wire decreases; thus, the mass block will rotate during the moving progress. As a result, the helical spring may be subjected to an axial spring moment M(t) due to the inertia of the mass block. It is worth noting that the spring force F(t) and moment M(t) enable the mass block to decelerate and move back. Then, as the helical spring moves upward, the light-driven contraction of the LCE wire is recovered in the dark zone, leading the mass block to rotate back. Meanwhile, the spring force of the helical spring decreases, which results in the mass block accelerating and moving downward again. Therefore, the mass block may oscillate up and down continuously over time, accompanied by the to-and-fro rotation. 

To describe the motion of the mass block, the axial displacement u(t) along the vertical direction is introduced, and the origin $O^{\prime }$ is selected at the lower end of the helical spring at the initial state. Meanwhile, an angular position θ(t) is introduced to denote the mass block rotating from the initial position. In the movement, the mass block is subjected to gravity mg, spring force F(t), spring moment M(t), damping force $F_{d}\left(t\right)$, and damping moment $M_{d}\left(t\right)$, as shown in Figure 2d. For simplicity, the damping force $F_{d}\left(t\right)$ and damping moment $M_{d}\left(t\right)$ are assumed to be proportional to the axial velocity du/dt and the angular velocity dθ/dt, respectively. Therefore, the corresponding nonlinear dynamic governing equation of mass block can be stated as follows:

(1) $$m\frac{d^{2}u}{dt^{2}}+\beta_{1}\frac{du}{dt}+F\left(t\right)-mg=0,$$

(2) $$J\frac{d^{2}\theta }{dt^{2}}+\beta_{2}\frac{d\theta }{dt}+M\left(t\right)=0,$$

where *g* is the gravitational acceleration, $\beta_{1}$ and $\beta_{2}$ are the translational damping coefficient and the rotational damping coefficient, respectively, $d^{2}u/dt^{2}$ and $d^{2}\theta /dt^{2}$ refer to the translational acceleration and rotational acceleration of mass block, respectively.

The initial conditions are stated as follows:

(3) $$\frac{du}{dt}={\dot{u}}_{0}\;\mathrm{and}\;\frac{d\theta \left(t\right)}{dt}={\dot{\theta }}_{0}\;\mathrm{at}\;t=0.$$

### 2.2. Helical Spring Model

During the motion of the mass block, the loaded helical spring with negligible mass is subjected to the combined spring force F(t) and spring moment M(t), and the equations relating the static deflections of helical springs to applied loads are discussed in detail by Wahl [68]. By following refs. [68,69], we assume that both the stretch of the helix centerline and the transverse shearing strains are negligible, and we can obtain the following:

(4) $$F\left(t\right)=\frac{EI_{z}\cos\alpha }{\left(1+\upsilon \right)R}\left(\frac{\sin\alpha \cos\alpha }{R}-\frac{\sin\alpha_{0}\cos\alpha_{0}}{R_{0}}\right)-\frac{EI_{z}\sin\alpha }{R}\left(\frac{\cos^{2}\alpha }{R}-\frac{\cos^{2}\alpha_{0}}{R_{0}}\right),$$

(5) $$M\left(t\right)=\frac{EI_{z}\sin\alpha }{\left(1+\upsilon \right)}\left(\frac{\sin\alpha \cos\alpha }{R}-\frac{\sin\alpha_{0}\cos\alpha_{0}}{R_{0}}\right)+EI_{z}\cos\alpha \left(\frac{\cos^{2}\alpha }{R}-\frac{\cos^{2}\alpha_{0}}{R_{0}}\right).$$

where the Young’s modulus and Poisson’s ratio are denoted by E and υ, respectively, and $I_{z}$ is the cross-sectional inertia moment of the LCE wire, which can be assumed to be a constant by ignoring the variation in cross-section for simplicity. Note that $EI_{z}$ represents the cross-sectional bending stiffness of the LCE wire.

To determine the spring force F(t) and spring moment M(t) in Equations (4) and (5), the current helix angle α(t) and coil radius R(t) should be calculated. From Figure 2c, the helix height h(t) is related to the current helix angle α(t) and the current length s(t) of the LCE wire by h(t)=s(t)sinα(t). Thus, the helix angle α(t) can be expressed as follows:

(6) α(t)=arcsin[h(t)/s(t)],

where

(7) $$h\left(t\right)=h_{0}+u\left(t\right).$$

Meanwhile, the angular position θ(t) of the mass block can be expressed by the current coil radius R(t) and current height h(t) of the helical spring as $\theta \left(t\right)=h\left(t\right)\mathrm{ctan}\alpha \left(t\right)/R\left(t\right)-h_{0}\mathrm{ctan}\alpha_{0}/R_{0}$, and thus we obtain the following:

(8) $$R\left(t\right)=\frac{\left(u+h_{0}\right)\mathrm{ctan}\alpha }{\theta +\frac{h_{0}}{R_{0}}\mathrm{ctan}\alpha_{0}}.$$

To figure out the helix angle α(t) in Equation (6) and the coil radius R(t) in Equation (8), the current length s(t) of the LCE wire is calculated in the following. Owing to neglecting the elastic deformation along the wire centerline, the length s(t) is only determined by light-driven contraction. It is worth noting that the light-driven contraction is inhomogeneous in an LCE wire. The Lagrangian arc coordinate system S in the reference state (Figure 2a) and the Eulerian coordinate system x in the current state (Figure 2b) are introduced to estimate the inhomogeneous deformation in the LCE wire. During the motion of the mass block, x=x(S,t) describes the instantaneous axial position of a material point in the LCE wire. Then, the light-driven contraction in the LCE wire denoted by $\varepsilon_{L}\left(S,t\right)$ is assumed to be proportional to the *cis-isomers* number fraction ɸ(S,t), i.e.,

(9) $$\varepsilon_{L}\left(S,t\right)=C_{0}ɸ\left(S,t\right),$$

where $C_{0}$ is the contraction coefficient. Thus, the current length s(t) of LCE wire can be expressed as follows:

(10) $$s\left(t\right)=\int_{0}^{s_{0}}\left(1-\varepsilon_{L}\right)dS=\int_{0}^{s_{0}}\left[1-C_{0}ɸ\left(S,t\right)\right]dS.$$

### 2.3. Dynamic LCE Model of the Wire

We employ the well-established dynamic LCE model proposed by Finkelmann et al. [58,70] to estimate the number fraction ɸ(S,t) of the *cis*-isomers of the LCE wire in Equation (10). Yu et al. found that UV or laser light with a wave length less than 400 nm can cause the trans-to-cis isomerization of LCE [71]. The number fraction ɸ(S,t) of the cis-isomers depends on the thermal excitation from trans to cis, the thermally driven relaxation from cis to trans, and the light driven relaxation from trans to cis. The thermal excitation from trans to cis is often considered to be negligible as opposed to the light-driven excitation [58,72]; thus, the number fraction ɸ(S,t) can be usually described by the governing equation as follows [32]:

(11) $$\frac{\partial ɸ\left(S,t\right)}{\partial t}=\eta_{0}I\left(S,t\right)\left[1-ɸ\left(S,t\right)\right]-\tau_{0}^{-1}ɸ\left(S,t\right),$$

where $\eta_{0}$ is the light absorption constant, and $\tau_{0}$ is the thermal relaxation time from *cis to trans*.

In Equation (11), the light intensity I(S,t) of material point S is determined by its current axial position x(S,t). The current light intensity is set as $I\left(S,t\right)=I_{0}$ for the part of the LCE wire under illumination, i.e., $x\left(S,t\right)>h_{0}$, and I(S,t)=0 for the part of the LCE wire in darkness, i.e., $x\left(S,t\right)\leq h_{0}$, as shown in Figure 2c. Considering the same current helix angle throughout the entire LCE helical spring, we can obtain the following:

(12) $$x\left(S,t\right)=\sin\alpha \int_{0}^{S}\left(1-\varepsilon_{L}\right)dS=\sin\alpha \int_{0}^{S}\left[1-C_{0}ɸ\left(S,t\right)\right]dS.$$

### 2.4. Nondimensionalization

To conveniently investigate the dynamics of the helical spring oscillator, the following dimensionless quantities are introduced: ${\bar{R}}_{0}=R_{0}/h_{0}$, ${\bar{E}}_{I}=EI_{z}\tau_{0}^{2}/mh_{0}^{3}$, ${\bar{\beta }}_{1}=\beta_{1}\tau_{0}/m$, ${\bar{\beta }}_{2}=\beta_{2}\tau_{0}/mh_{0}^{2}$, $\bar{g}=g\tau_{0}^{2}/h_{0}$, $\bar{J}=J/mh_{0}^{2}$, ${\bar{I}}_{0}=I_{0}\eta_{0}\tau_{0}$, ${\bar{s}}_{0}=s_{0}/h_{0}$, $\bar{s}=s/h_{0}$, $\bar{t}=t/\tau_{0}$, $\bar{I}=I\eta_{0}\tau_{0}$, $\bar{R}=R/h_{0}$, $\bar{u}=u/h_{0}$, $\bar{S}=S/h_{0}$, $\bar{x}=x/h_{0}$, $\bar{F}=F\tau_{0}^{2}/mh_{0}$, and $\bar{M}=M\tau_{0}^{2}/mh_{0}^{2}$. The governing equations in Equations (1)–(6), (8), and (10) can be rewritten as follows:

(13) $$\frac{d^{2}\bar{u}}{d{\bar{t}}^{2}}+{\bar{\beta }}_{1}\frac{d\bar{u}}{d\bar{t}}+\bar{F}\left(\bar{t}\right)-\bar{g}=0,$$

(14) $$\bar{J}\frac{d^{2}\theta }{d{\bar{t}}^{2}}+{\bar{\beta }}_{2}\frac{d\theta }{d\bar{t}}+\bar{M}\left(\bar{t}\right)=0,$$

with initial conditions as follows:

(15) $$\frac{d\bar{u}\left(\bar{t}\right)}{d\bar{t}}={\dot{\bar{u}}}_{0}\;\mathrm{and}\;\frac{d\theta \left(\bar{t}\right)}{d\bar{t}}={\dot{\bar{\theta }}}_{0}\;\mathrm{at}\;\bar{t}=0,$$

where $\bar{F}\left(\bar{t}\right)$ and $\bar{M}\left(\bar{t}\right)$ can be calculated as the following:

(16) $$\bar{F}\left(\bar{t}\right)=\frac{{\bar{E}}_{I}\cos\alpha }{\left(1+\upsilon \right)\bar{R}}\left[\frac{\sin\alpha \cos\alpha }{\bar{R}}-\frac{\sin\alpha_{0}\cos\alpha_{0}}{{\bar{R}}_{0}}\right]-\frac{{\bar{E}}_{I}\sin\alpha }{\bar{R}}\left(\frac{\cos^{2}\alpha }{\bar{R}}-\frac{\cos^{2}\alpha_{0}}{{\bar{R}}_{0}}\right)$$

(17) $$\bar{M}\left(\bar{t}\right)=\frac{{\bar{E}}_{I}}{\left(1+\upsilon \right)}\sin\alpha \left[\frac{\sin\alpha \cos\alpha }{\bar{R}}-\frac{\sin\alpha_{0}\cos\alpha_{0}}{{\bar{R}}_{0}}\right]+{\bar{E}}_{I}\cos\alpha \left[\frac{\cos^{2}\alpha }{\bar{R}}-\frac{\cos^{2}\alpha_{0}}{{\bar{R}}_{0}}\right]$$

where

(18) $$\alpha \left(\bar{t}\right)=\arcsin\left[\left(\bar{u}+1\right)/\bar{s}\right],$$

(19) $$\bar{R}\left(\bar{t}\right)=\frac{\left(\bar{u}+1\right)\mathrm{ctan}\alpha }{\theta +\frac{1}{{\bar{R}}_{0}}\mathrm{ctan}\alpha_{0}},$$

(20) $$\bar{s}\left(\bar{t}\right)=\int_{0}^{1/\sin\alpha_{0}}\left[1-C_{0}ɸ\left(\bar{S},\bar{t}\right)\right]d\bar{S}.$$

Meanwhile, $ɸ\left(\bar{S},\bar{t}\right)$ can be calculated from Equation (11) as follows:

(21) $$\frac{\partial ɸ\left(\bar{S},\bar{t}\right)}{\partial \bar{t}}=\bar{I}\left(\bar{S},\bar{t}\right)\left[1-ɸ\left(\bar{S},\bar{t}\right)\right]-ɸ\left(\bar{S},\bar{t}\right),$$

where $\bar{I}\left(\bar{S},\bar{t}\right)={\bar{I}}_{0}$ for $\bar{x}\left(\bar{S},\bar{t}\right)>1$; otherwise, $\bar{I}\left(\bar{S},\bar{t}\right)=0$. It is worth noting that the dimensionless instantaneous material position $\bar{x}\left(\bar{S},\bar{t}\right)$ can be determined by Equation (12) as follows:

(22) $$\bar{x}\left(\bar{S},\bar{t}\right)=\sin\alpha \int_{0}^{\bar{S}}\left[1-C_{0}ɸ\left(\bar{S},\bar{t}\right)\right]d\bar{S}$$

Equations (13), (14), and (21) govern the self-tension–torsion of the LCE helical spring oscillator under steady illumination, in which the time-dependent number fraction of *cis*-isomers in the LCE wire is coupled with the displacement of the mass block. The fourth-order Runge–Kutta method is utilized to solve these complex differential equations with variable coefficients, and the numerical calculations were carried out using Matlab software. For the given number fraction ${ɸ}_{i}$ of *cis*-isomers in the LCE wire, the axial displacement ${\bar{u}}_{i}$ and angular position $\theta_{i}$ of the mass block at time ${\bar{t}}_{i}$, the current length ${\bar{s}}_{i}$ of the LCE wire, the current helix angle $\alpha_{i}$, and coil radius ${\bar{R}}_{i}$ of the LCE helical spring can be determined from Equations (18)–(20). Subsequently, the current spring force ${\bar{F}}_{i}$ and spring moment ${\bar{M}}_{i}$ can be further calculated from Equations (16) and (17), respectively. Following that, the axial displacement ${\bar{u}}_{i+1}$ and angular position $\theta_{i+1}$ of the mass block at time ${\bar{t}}_{i+1}$ can be calculated from Equations (13) and (14). Meanwhile, the current axial position ${\bar{x}}_{i}\left(\bar{S},{\bar{t}}_{i}\right)$ of material point $\bar{S}$ is determined to estimate the light intensity $\bar{I}\left(\bar{S},{\bar{t}}_{i}\right)$ from Equation (22), and then the number fraction ${ɸ}_{i+1}$ of *cis*-isomers in the LCE wire can be calculated from Equation (21). In brief, through iteration, the self-oscillating combined tension and torsion of the LCE helical spring oscillator can be numerically obtained for given parameters $C_{0}$, ${\bar{I}}_{0}$, $\bar{g}$, $\bar{J}$, ${\bar{\beta }}_{1}$, ${\bar{\beta }}_{2}$, $\alpha_{0}$, ${\bar{R}}_{0}$, ${\bar{E}}_{I}$, υ, ${\dot{\bar{u}}}_{0}$, and ${\dot{\bar{\theta }}}_{0}$. 

## 3. Two Motion Regimes and Mechanism of Self-Oscillation

In this section, based on the governing Equations (13), (14), and (21), we first present two typical motion regimes of the helical spring oscillator, namely the static regime and the self-tension–torsion regime. Then, the corresponding mechanism of self-tension–torsion is elucidated in detail.

### 3.1. Two Motion Regimes

To investigate the self-tension–torsion of the helical spring oscillator under steady illumination, we should first determine the specific values of those dimensionless parameters in the model. The existing experiments [23,73,74] provide us the typical material properties and geometric parameters, as listed in Table 1. Table 2 shows the corresponding dimensionless parameters. In the following, these values of parameters are used to study the self-tension–torsion of the helical spring oscillator under steady illumination. In the calculation, we fix the Poisson’s ratio of material as υ=0.5, the helix angle of the stress-free helical spring as $\alpha_{0}=0.03$, and the initial conditions as ${\dot{\bar{u}}}_{0}=0$ and ${\dot{\bar{\theta }}}_{0}=0$.

**Figure 3:**
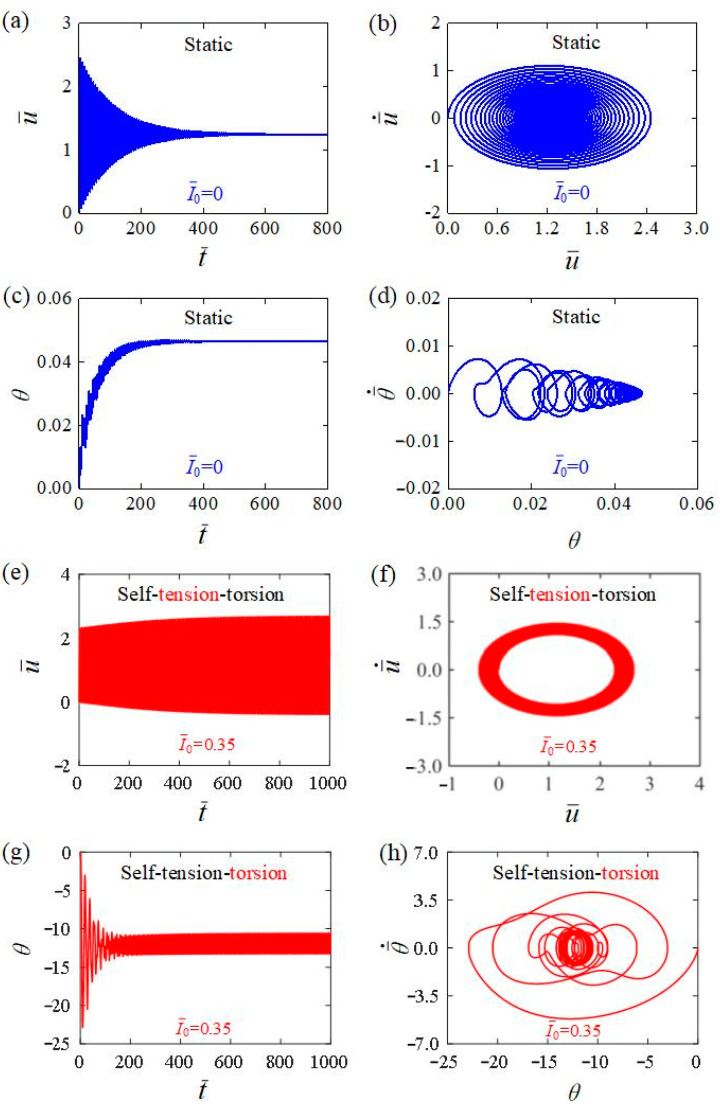
Time histories and phase trajectories for two motion regimes of the helical spring oscillator. (**a**–**d**) The static regime with ${\bar{I}}_{0}=0$; (**e**–**h**) the self-tension–torsion regime with ${\bar{I}}_{0}=0.35$. The other parameters are $C_{0}=0.3$, ${\bar{\beta }}_{1}=0.02$, ${\bar{\beta }}_{2}=0.005$, ${\bar{E}}_{I}=0.4$, ${\bar{R}}_{0}=0.1$, $\bar{J}=0.1$, and $\bar{g}=1$. Upon exposure to steady illumination, the helical spring oscillator possesses two typical motion regimes, namely the static regime and the self-tension–torsion regime.

From Equations (13) to (22), the time histories and phase trajectories of the light-powered motion of helical spring oscillator can be obtained, among which the cases for ${\bar{I}}_{0}=0$ and ${\bar{I}}_{0}=0.35$ are plotted in Figure 3. In the calculation, we set the other parameters as $C_{0}=0.3$, ${\bar{\beta }}_{1}=0.02$, ${\bar{\beta }}_{2}=0.005$, ${\bar{E}}_{I}=0.4$, ${\bar{R}}_{0}=0.1$, $\bar{J}=0.1$, and $\bar{g}=1$. For ${\bar{I}}_{0}=0$ shown in Figure 3a–d, the amplitudes for both the translation $\bar{u}$ and the rotation θ exhibit a gradual decrease with time due to the damping dissipation, and the helical spring oscillator eventually becomes stationary at the equilibrium position, which is named as the static regime. For ${\bar{I}}_{0}=0.35$ shown in Figure 3e–h, the mass block initially vibrates from its initial position, then the oscillating amplitudes for both the translation $\bar{u}$ and the rotation θ progressively increase over time, then finally remain constant. When exposed to steady illumination, the helical spring oscillator eventually presents a continuous periodic oscillatory motion that combines tension and torsion, which can be named as the self-tension–torsion regime. The steady-state time histories of translation and rotation are consistent with the experimental results in Figure 1 [51].

### 3.2. Mechanisms of the Self-Tension–Torsion

**Figure 4:**
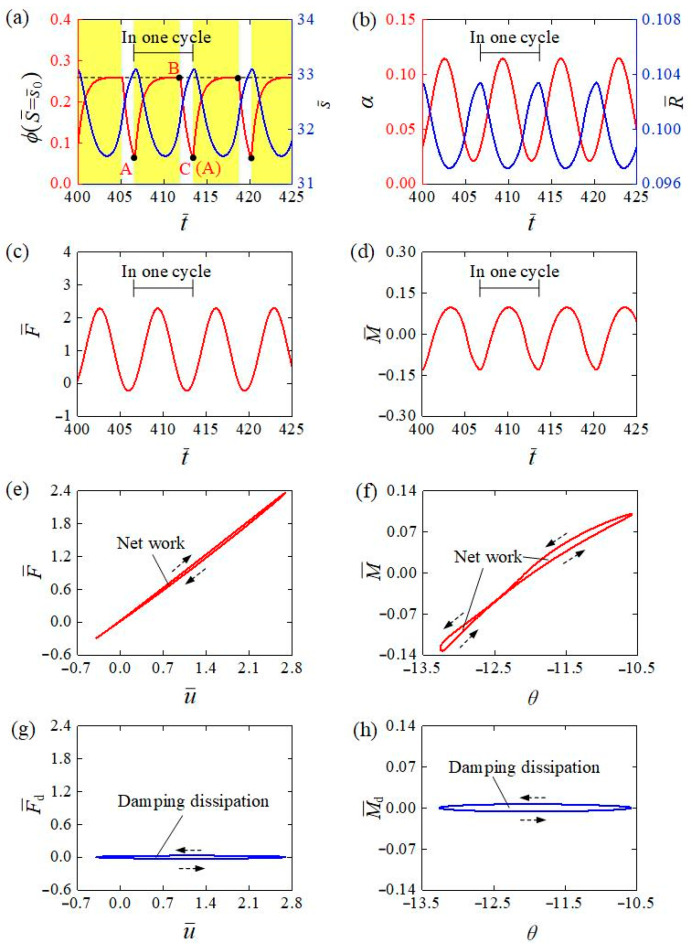
Mechanism of the self-tension–torsion for the typical case in Figure 3e–h. (**a**) The time evolutions of *cis*-isomers number fraction at $\bar{S}={\bar{s}}_{0}$ and length of LCE wire. (**b**) The time evolutions of helix angle α and coil radius $\bar{R}$ of helical spring. (**c**) The time evolution spring force of helical spring. (**d**) The time evolution of spring moment of helical spring. (**e**) The dependence between spring force and axial displacement in one cycle of the self-tension–torsion. (**f**) The dependence between spring moment and angular position in one cycle of the self-tension–torsion. (**g**) The dependence between damping force and axial displacement in one cycle of the self-tension–torsion. (**h**) The dependence between damping moment and angular position in one cycle of the self-tension–torsion. The area enclosed by the circles in (**e**,**f**) respectively represent the net work done by the spring force and moment, which compensate for the damping dissipation to maintain the self-tension–torsion.

**Figure 5:**
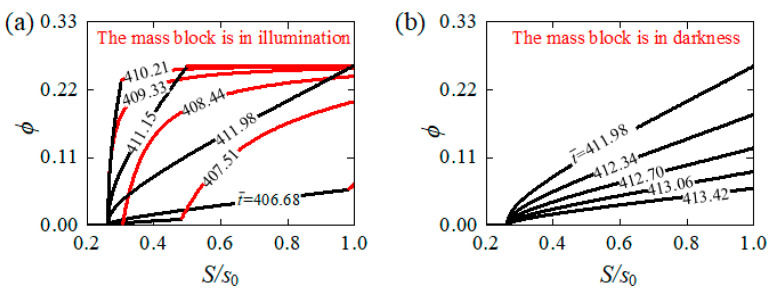
Evolution of the inhomogeneous number fraction of *cis*-isomers in LCE wire for the cases in Figure 3e–h. (**a**) The mass block is under illumination. (**b**) The mass block is in the dark. The red curve depicts the part of the LCE wire under illumination, while the black curve depicts the part in the dark. The inhomogeneous number fraction of *cis*-isomers in LCE wire periodically varies over time.

**Figure 6:**
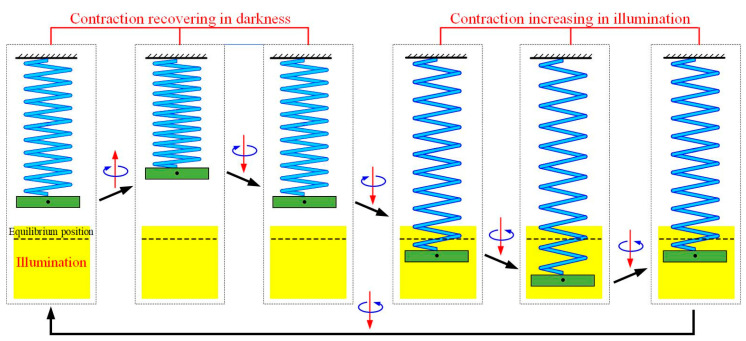
The snapshots in one cycle of self-tension–torsion for the helical spring oscillator corresponding to Figure 3e–h. The red and blue arrows represent the translational direction and rotational direction of mass block, respectively. Upon exposure to steady illumination, the helical spring oscillator presents a continuous periodic self-tension–torsion owing to the periodically varying light-driven contraction of LCE wire.

To figure out the mechanism of the self-tension–torsion of the helical spring oscillator under steady illumination, Figure 4 illustrates several vital physical quantities of the LCE wire for the typical case in Figure 3e–h. Figure 4a shows the number fraction of *cis*-isomers ɸ at the representative material point $\bar{S}={\bar{s}}_{0}$ versus time (red curve), and the current length $\bar{s}$ of the LCE wire versus time (blue curve). When the material point moves into the illumination (see the yellow shaded areas in Figure 4a), the red curve A-B reveals that the ɸ gradually increases over time and attains a limit value, while the red curve B-C displays the gradual decrease in ɸ in the dark. The inhomogeneous distribution of the number fraction ɸ in the LCE wire is further illustrated in Figure 5a,b for the mass block in illumination and in darkness, respectively. From the snapshots depicted in Figure 6, four stages constitute one cycle in the oscillatory process, namely the downward movement of the mass block in illumination, the upward movement of the mass block in illumination, the upward movement of the mass block in darkness and the downward movement of the mass block in darkness. In Figure 5, the part of the LCE wire in illumination is depicted by the red curve, while the other part in darkness is depicted by the black curve. In general, the number fraction of *cis*-isomers increases over time for the part of the LCE wire in illumination, while this decreases for the other part in darkness, leading to the same periodic variation in the length $\bar{s}$ of the LCE wire, as shown in Figure 4a.

Owing to the periodically varying number fraction of *cis*-isomers ɸ, the helix angle α, coil radius $\bar{R}$, spring force $\bar{F}$, and spring moment $\bar{M}$ also exhibit periodic behaviors, as shown in Figure 4b–d. Figure 4e describes the dependence of the spring force $\bar{F}$ on the axial displacement $\bar{u}$ for the case shown in Figure 3e–h. During the upward oscillating process of the mass block in darkness, the spring force $\bar{F}$ presents a significant decrease due to the joint action of the decreasing height of the helical spring and the increasing length of the LCE wire, along with the contraction recovery in darkness. When the mass block moves downward in darkness, the spring force $\bar{F}$ turns to increase due to the joint action of the increasing height of the helical spring and the increasing length of the LCE wire, along with the contraction recovery in darkness. During the downward oscillating process of the mass block in illumination, the spring force $\bar{F}$ has a significant increase due to the joint action of the increasing height of the helical spring and the contraction of the increasing part of the LCE wire in light. When the mass block moves upward in illumination, the spring force $\bar{F}$ decreases due to the decreasing height of the helical spring and the contraction recovery of the increasing part of the LCE wire in darkness. Finally, the plot of the spring force $\bar{F}$ versus axial displacement $\bar{u}$ forms a counterclockwise closed circle. The area surrounded by the circle represents the net work $W_{\mathrm{Fs}}$ done by the spring force, which is calculated as 0.138. Meanwhile, Figure 4f reveals the dependence of the spring moment $\bar{M}$ on the angular position θ, where their relation curve forms two counterclockwise closed circles in one cycle of self-tension–torsion. Equations (17)–(19) could be the explanation for this result. The spring moment $\bar{M}$ is jointly determined by the length $\bar{s}$ of the LCE wire, the helix angle α, and coil radius $\bar{R}$ of the helical spring, while the angular position θ is largely dominated by the periodically varying length $\bar{s}$ of the LCE wire from Equation (8) and Figure 4b. The area surrounded by the circles in Figure 4f represents the positive net work $W_{\mathrm{Ms}}$ done by the spring moment, which is calculated to be 0.0263.

Similarly, Figure 4g depicts the relationship curve between the damping force ${\bar{F}}_{d}$ and axial displacement $\bar{u}$ in one self-tension–torsion cycle, which forms a counterclockwise closed circle, and the negative net work ${\bar{M}}_{\mathrm{Fd}}$ done by the damping force ${\bar{F}}_{d}$ can be calculated to be 0.138. At the same time, Figure 4h plots the curve of the damping moment ${\bar{M}}_{d}$ versus angular position θ in one self-tension–torsion cycle, which also forms a counterclockwise closed circle, and the negative net work ${\bar{M}}_{\mathrm{Md}}$ done by the damping moment ${\bar{M}}_{d}$ can be calculated to be 0.0263. In summary, $W_{\mathrm{Fs}}$ in Figure 4e is equal to $W_{\mathrm{Fd}}$ in Figure 4g, while $W_{\mathrm{Ms}}$ in Figure 4f is also equal to $W_{\mathrm{Md}}$ in Figure 4h, indicating that the energy dissipation of the damping $W_{\mathrm{Fd}}$ and $W_{\mathrm{Md}}$ are fully compensated by the $W_{\mathrm{Fs}}$ and $W_{\mathrm{Ms}}$, respectively.

## 4. Effects of System Parameters on the Self-Tension–Torsion

In this section, the influence of eight dimensionless system parameters, including $C_{0}$, ${\bar{I}}_{0}$, $\bar{g}$, $\bar{J}$, ${\bar{\beta }}_{1}$, ${\bar{\beta }}_{2}$, ${\bar{R}}_{0}$, and ${\bar{E}}_{I}$, on the triggering conditions, frequency, and amplitude of self-tension–torsion, are investigated in detail. In this study, the dimensionless oscillatory frequency and amplitude of axial displacement are respectively denoted by $f_{u}$ and $A_{u}$, while the frequency and amplitude of the angular position are respectively denoted by $f_{\theta }$ and $A_{\theta }$.

### 4.1. Influence of the Light Intensity

**Figure 7:**
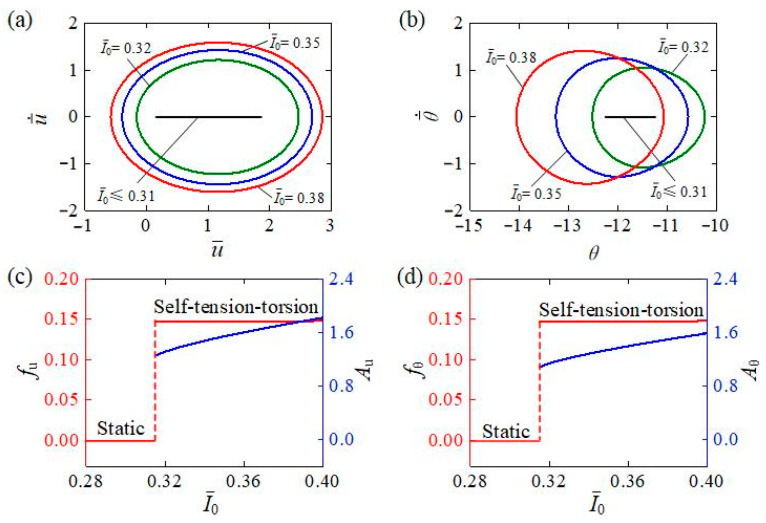
The influence of light intensity on the self-tension–torsion, for $C_{0}=0.3$, ${\bar{\beta }}_{1}=0.02$, ${\bar{\beta }}_{2}=0.005$, ${\bar{E}}_{I}=0.4$, ${\bar{R}}_{0}=0.1$, $\bar{J}=0.1$, and $\bar{g}=1$. (**a**) Limit cycles for the translation. (**b**) Limit cycles for the rotation. (**c**) Frequency and amplitude of the translation under different light intensities. (**d**) Frequency and amplitude of the rotation under different light intensities. The amplitudes of both translation and rotation tend to rise with increasing light intensity, while the frequencies of both translation and rotation are almost independent of light intensity.

Figure 7 describes the influence of light intensity ${\bar{I}}_{0}$ on the self-tension–torsion of the helical spring oscillator. In the calculation, we set $C_{0}=0.3$, ${\bar{\beta }}_{1}=0.02$, ${\bar{\beta }}_{2}=0.005$, ${\bar{E}}_{I}=0.4$, ${\bar{R}}_{0}=0.1$, $\bar{J}=0.1$, and $\bar{g}=1$. A critical light intensity was determined to be around 0.31 for triggering the self-tension–torsion. When ${\bar{I}}_{0}\leq 0.31$, the helical spring oscillator always rests at the static equilibrium position, indicating the static regime, while the self-tension–torsion can be triggered at ${\bar{I}}_{0}=0.32$, ${\bar{I}}_{0}=0.35$, and ${\bar{I}}_{0}=0.38$ with limit cycles for the translation and rotation depicted in Figure 7a,b, respectively. The light intensity ${\bar{I}}_{0}$ has the same effect on the frequency and amplitude for translation and rotation, as depicted in Figure 7c,d. The amplitudes of both translation and rotation tend to rise with the increasing light intensity ${\bar{I}}_{0}$, while the frequencies of both translation and rotation are almost independent of the light intensity. It is worth noting that the frequency of translation is always the same as that of rotation, which is a common characteristic for other system parameters in this study. Absorbing light energy to convert into mechanical energy accounts for the generation of oscillatory motion. As the light intensity increases, more of the light energy will be converted to mechanical energy, so one can improve the energy absorption of the self-tension–torsion of the helical spring oscillator by intensifying the light.

### 4.2. Influence of the Contraction Coefficient

**Figure 8:**
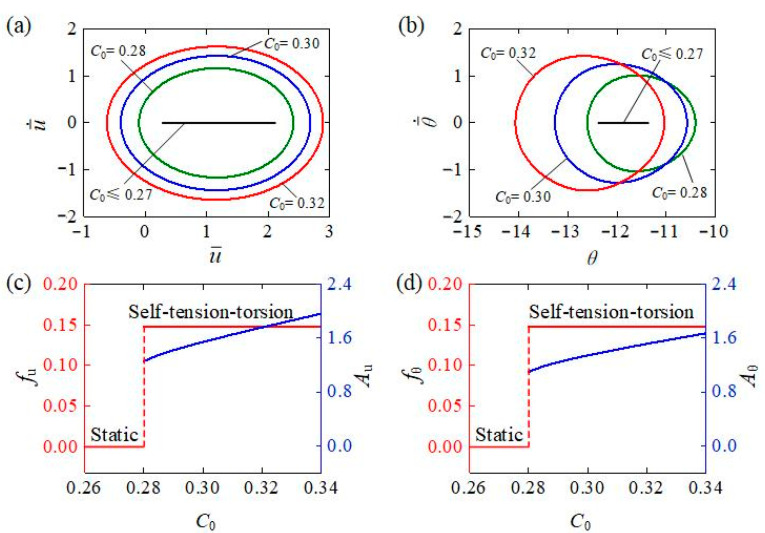
The influence of contraction coefficient on the self-tension–torsion, for ${\bar{I}}_{0}=0.35$, ${\bar{\beta }}_{1}=0.02$, ${\bar{\beta }}_{2}=0.005$, ${\bar{E}}_{I}=0.4$, ${\bar{R}}_{0}=0.1$, $\bar{J}=0.1$, and $\bar{g}=1$. (**a**) Limit cycles for the translation. (**b**) Limit cycles for the rotation. (**c**) Frequency and amplitude of the translation under different contraction coefficients. (**d**) Frequency and amplitude of the rotation under different contraction coefficients. Contraction coefficient has almost no impact on the frequency, while the amplitudes of both translation and rotation exhibit upward trends with increasing contraction coefficient.

Figure 8 describes the influence of the contraction coefficient $C_{0}$ on the self-tension–torsion of the helical spring oscillator. In the calculation, we set $C_{0}=0.3$, ${\bar{\beta }}_{1}=0.02$, ${\bar{\beta }}_{2}=0.005$, ${\bar{E}}_{I}=0.4$, ${\bar{R}}_{0}=0.1$, $\bar{J}=0.1$, and $\bar{g}=1$. Similarly, the critical contraction coefficient was calculated to be approximately 0.27 for triggering the self-tension–torsion, while the helical spring oscillator stays at the static equilibrium position for $C_{0}\leq 0.27$. For different contraction coefficients $C_{0}=0.28$, $C_{0}=0.30$, and $C_{0}=0.32$, the self-tension–torsion can be triggered and the respective limit circles for the translation and the rotation are shown in Figure 8a,b. As described in Figure 8c,d, the contraction coefficient $C_{0}$ has almost no impact on the frequency, while the amplitudes of both translation and rotation exhibit upward trends with increasing $C_{0}$. The result suggests that the efficiency of conversion from light to mechanical energy can be improved through increasing the contraction coefficient of the LCE material.

### 4.3. Influence of the Translational Damping Coefficient

**Figure 9:**
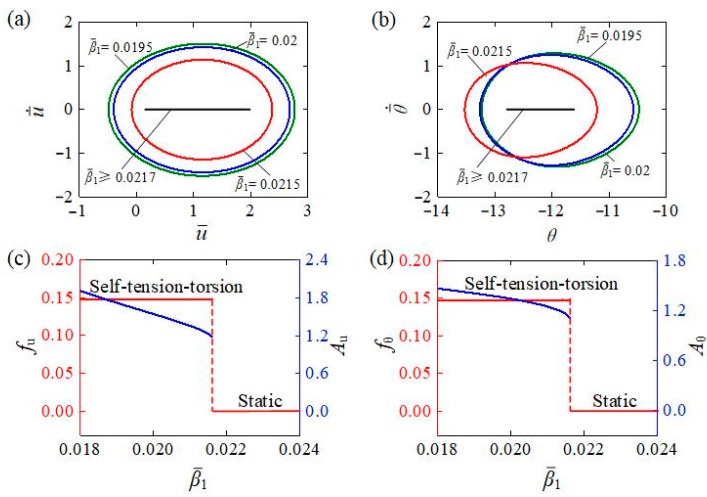
The influence of translational damping coefficient on the self-tension–torsion for ${\bar{I}}_{0}=0.35$, $C_{0}=0.3$, ${\bar{\beta }}_{2}=0.005$, ${\bar{E}}_{I}=0.4$, ${\bar{R}}_{0}=0.1$, $\bar{J}=0.1$, and $\bar{g}=1$. (**a**) Limit cycles for the translation. (**b**) Limit cycles for the rotation. (**c**) Frequency and amplitude of the translation under different translational damping coefficients. (**d**) Frequency and amplitude of the rotation under different translational damping coefficients. The increase in the translational damping coefficient leads to a decline in both translational and rotational amplitudes, while the translational and rotational frequencies remain almost unaffected.

The influence of the translational damping coefficient ${\bar{\beta }}_{1}$ on the self-tension–torsion is depicted in Figure 9, including the limit cycles for translation and rotation under different translational damping coefficients (Figure 9a,b) and the dependences of the amplitude and frequency for different translational damping coefficients (Figure 9c,d). The other parameters were set as ${\bar{I}}_{0}=0.35$, $C_{0}=0.3$, ${\bar{\beta }}_{2}=0.005$, ${\bar{E}}_{I}=0.4$, ${\bar{R}}_{0}=0.1$, $\bar{J}=0.1$, and $\bar{g}=1$. A critical translational damping coefficient ${\bar{\beta }}_{1}$ valued at approximately 0.0217 exists to trigger the self-tension–torsion. For ${\bar{\beta }}_{1}\geq 0.0217$, the damping dissipation is too large for the light energy input to compensate for; thus, it exhibits the static regime. In contrast, for ${\bar{\beta }}_{1}=0.0195$, ${\bar{\beta }}_{1}=0.02$, and ${\bar{\beta }}_{1}=0.0215$, the self-tension–torsion can be triggered. The increase in the translational damping coefficient leads to a decline in both the translational and rotational amplitudes, while the translational and rotational frequencies remain almost unaffected. The explanation may be that the larger the translational damping coefficient is, the more the energy is dissipated, and therefore the smaller the amplitudes become. As a result, proper reduction of the translational damping coefficient is one of the most effective measures to achieve significant energy harvesting in engineering the self-tension–torsion helical spring oscillator.

### 4.4. Influence of the Rotational Damping Coefficient

**Figure 10:**
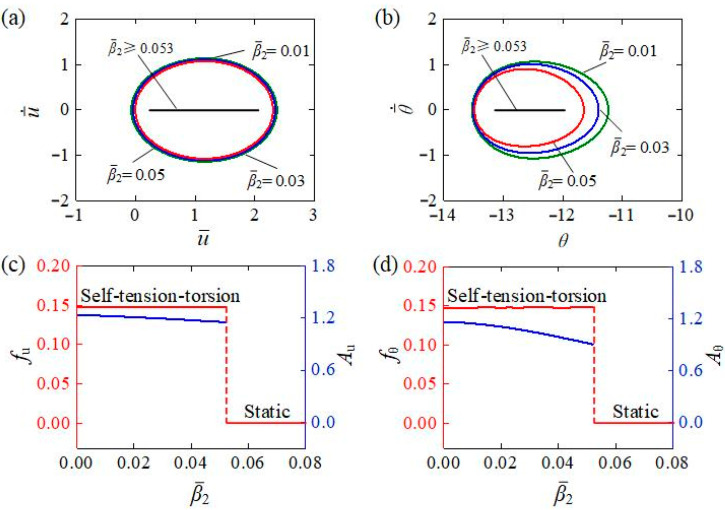
The influence of rotational damping coefficient on the self-tension–torsion for ${\bar{I}}_{0}=0.35$, $C_{0}=0.3$, ${\bar{\beta }}_{1}=0.0215$, ${\bar{E}}_{I}=0.4$, ${\bar{R}}_{0}=0.1$, $\bar{J}=0.1$, and $\bar{g}=1$. (**a**) Limit cycles for the translation. (**b**) Limit cycles for the rotation. (**c**) Frequency and amplitude of the translation under different rotational damping coefficients. (**d**) Frequency and amplitude of the rotation under different rotational damping coefficients. As the rotational damping coefficient increases, the amplitudes of self-tension–torsion for both translation and rotation decrease, but the frequencies for both translation and rotation are still virtually unaffected.

Figure 10 represents the effect of the rotational damping coefficient ${\bar{\beta }}_{2}$ on the self-tension–torsion, for ${\bar{I}}_{0}=0.35$, $C_{0}=0.3$, ${\bar{\beta }}_{1}=0.0215$, ${\bar{E}}_{I}=0.4$, ${\bar{R}}_{0}=0.1$, $\bar{J}=0.1$, and $\bar{g}=1$. Figure 10a,b separately plot the limit cycles of the translation and rotation under different rotational damping coefficients. The results indicate that there exists a critical rotational damping coefficient ${\bar{\beta }}_{2}$ to trigger the self-tension–torsion, which was numerically determined to be approximately 0.053. This is because the energy input to the system cannot compensate for the damping dissipation for ${\bar{\beta }}_{2}\geq 0.053$. For ${\bar{\beta }}_{2}=0.01$, ${\bar{\beta }}_{2}=0.03$, and ${\bar{\beta }}_{2}=0.05$, the self-tension–torsion can be triggered. The dependences of amplitude and frequency on the rotational damping coefficient are also shown in Figure 10c,d, respectively. As the rotational damping coefficient increases, the amplitudes of the self-tension–torsion for both translation and rotation decrease, but the frequencies for both translation and rotation are still virtually unaffected. These results can also be explained by the energy competition between light energy input and damping dissipation. The larger the rotational damping coefficient is, the more the energy dissipation generated, and thus the smaller the amplitudes become. Similarly, reducing the rotational damping coefficient in an appropriate manner is also one of the effective measures to achieve significant energy harvesting for the self-tension–torsion helical spring oscillator.

### 4.5. Influence of the Coil Radius

**Figure 11:**
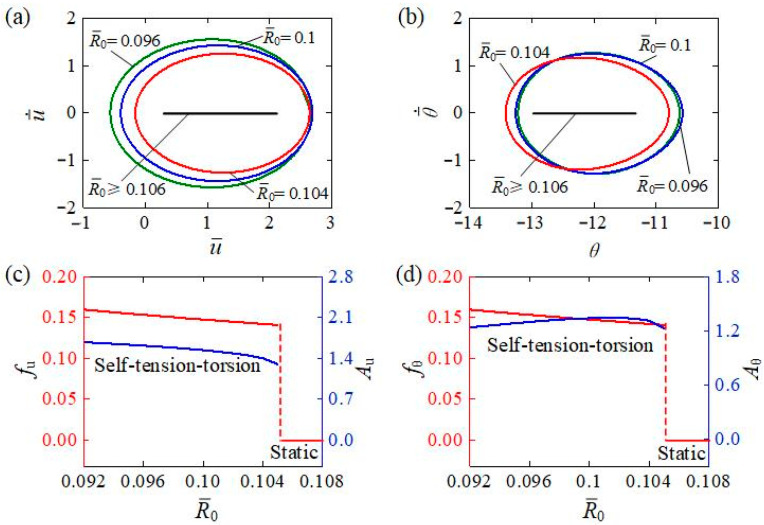
The influence of coil radius on the self-tension–torsion for ${\bar{I}}_{0}=0.35$, $C_{0}=0.3$, ${\bar{\beta }}_{1}=0.02$, ${\bar{\beta }}_{2}=0.005$, ${\bar{E}}_{I}=0.4$, $\bar{J}=0.1$, and $\bar{g}=1$. (**a**) Limit cycles for the translation. (**b**) Limit cycles for the rotation. (**c**) Frequency and amplitude of the translation under different coil radii. (**d**) Frequency and amplitude of the rotation under different coil radii. Increasing the coil radius, frequency, and amplitude of translation decreases owing to the fact that increasing the coil radius softens the helical spring. Meanwhile, the amplitude of rotation increases slightly at first, and then decreases as the coil radius increases.

Figure 11 presents how the coil radius ${\bar{R}}_{0}$ affects the self-tension–torsion for ${\bar{I}}_{0}=0.35$, $C_{0}=0.3$, ${\bar{\beta }}_{1}=0.02$, ${\bar{\beta }}_{2}=0.005$, ${\bar{E}}_{I}=0.4$, $\bar{J}=0.1$, and $\bar{g}=1$. From the limit cycles of translation and rotation under different coil radii ${\bar{R}}_{0}$ plotted in Figure 11a,b, there also exists a critical coil radius ${\bar{R}}_{0}$ for triggering the self-tension–torsion, which was numerically determined to be approximately 0.106. The helical spring oscillator for ${\bar{R}}_{0}\geq 0.106$ is in the static regime, while the one for ${\bar{R}}_{0}<0.106$ is in the self-tension–torsion regime. This is because the larger the coil radius is, the softer the helical spring is, and the less the strain energy generated. The dependences of amplitude and frequency on the coil radius are also illustrated in Figure 11c,d, respectively. When the coil radius ${\bar{R}}_{0}$ increases, the frequency and amplitude of translation decreases, owing to the fact that increasing the coil radius will soften the helical spring. Meanwhile, the amplitude of rotation increases slightly at first, and then decreases as the coil radius increases. This complex relationship results from the nonmonotonic dependences of the spring force and moment on ${\bar{R}}_{0}$ during the oscillatory progress expressed in Equations (16), (17), and (19). This suggests that appropriate adjustments of ${\bar{R}}_{0}$ within a certain range can modulate the oscillatory amplitude and the energy conversion efficiency from light to mechanical energy in engineering applications.

### 4.6. Influence of the Bending Stiffness

**Figure 12:**
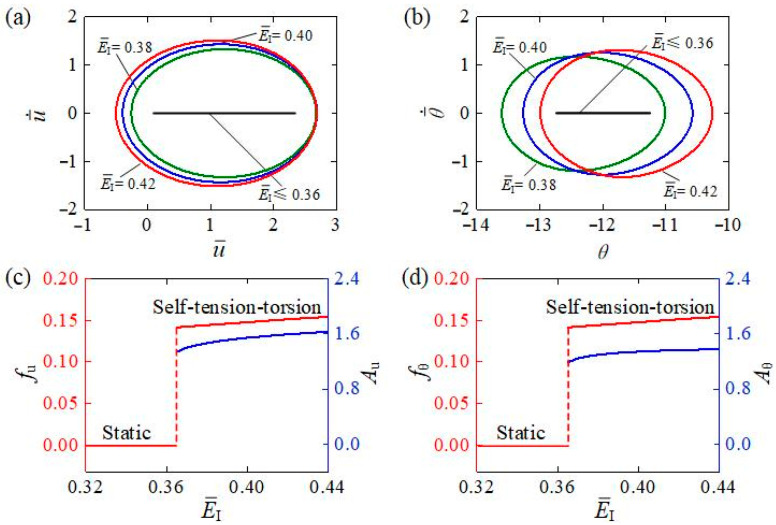
The influence of bending stiffness on the self-tension–torsion for ${\bar{I}}_{0}=0.35$, $C_{0}=0.3$, ${\bar{\beta }}_{1}=0.02$, ${\bar{\beta }}_{2}=0.005$, ${\bar{R}}_{0}=0.1$, $\bar{J}=0.1$, and $\bar{g}=1$. (**a**) Limit cycles for the translation. (**b**) Limit cycles for the rotation. (**c**) Frequency and amplitude of the translation under different bending stiffnesses. (**d**) Frequency and amplitude of the rotation under different bending stiffnesses. Along with the increasing bending stiffness, both amplitudes and frequencies of the self-tension–torsion for translation and rotation increase.

Figure 12 reveals the effect of bending stiffness ${\bar{E}}_{I}$ on the self-tension–torsion for ${\bar{I}}_{0}=0.35$, $C_{0}=0.3$, ${\bar{\beta }}_{1}=0.02$, ${\bar{\beta }}_{2}=0.005$, ${\bar{R}}_{0}=0.1$, $\bar{J}=0.1$, and $\bar{g}=1$. Figure 12a,b respectively plot the limit cycles of translation and rotation for different bending stiffnesses. As expected, the critical bending stiffness ${\bar{E}}_{I}$ to trigger the self-tension–torsion was numerically determined to be approximately 0.36. We attribute this to the fact that the energy input to the system cannot compensate for the damping dissipation for ${\bar{E}}_{I}\leq 0.36$. For ${\bar{E}}_{I}=0.38$, ${\bar{E}}_{I}=0.40$, and ${\bar{E}}_{I}=0.42$, the self-tension–torsion can be triggered. Figure 12c,d depict the dependences of the amplitude and frequency on bending stiffness, respectively. Along with the increasing bending stiffness, both the amplitudes and frequencies of the self-tension–torsion for translation and rotation increase. This result is also attributed to the energy competition between light energy input and damping dissipation. The larger the bending stiffness is, the more the strain energy generated. Consequently, increasing the bending stiffness is also among the effective measures to achieve significant energy harvesting for the self-tension–torsion LCE helical spring oscillator.

### 4.7. Influence of the Gravitational Acceleration

**Figure 13:**
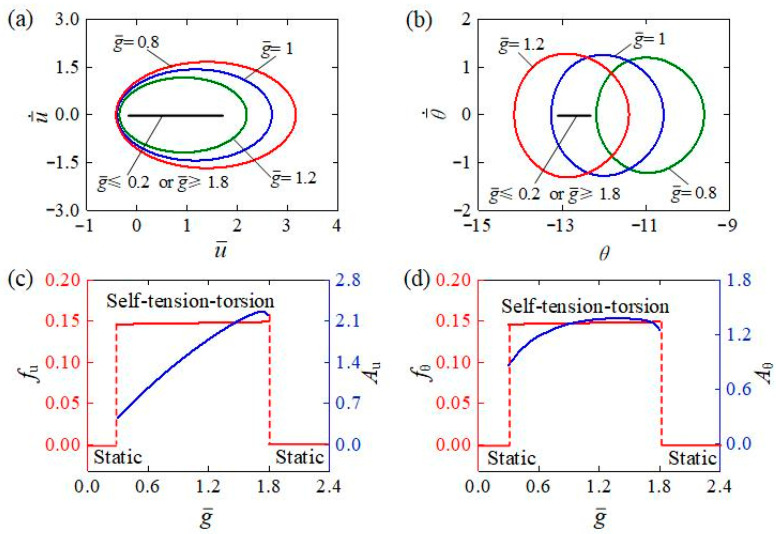
The effect of gravitational acceleration on the self-tension–torsion for ${\bar{I}}_{0}=0.35$, $C_{0}=0.3$, ${\bar{\beta }}_{1}=0.0215$, ${\bar{\beta }}_{2}=0.005$, ${\bar{E}}_{I}=0.4$, ${\bar{R}}_{0}=0.1$, $\bar{J}=0.1$, and $\bar{g}=1$. (**a**) Limit cycles for the translation. (**b**) Limit cycles for the rotation. (**c**) Frequency and amplitude of the translation under different gravitational accelerations. (**d**) Frequency and amplitude of the rotation under different gravitational accelerations. The amplitudes of self-tension–torsion for both translation and rotation first increase and then decrease with the increasing gravitational acceleration, while the frequencies slightly decrease.

Figure 13 demonstrates what effect the gravitational acceleration has on the self-tension–torsion for ${\bar{I}}_{0}=0.35$, $C_{0}=0.3$, ${\bar{\beta }}_{1}=0.0215$, ${\bar{\beta }}_{2}=0.005$, ${\bar{E}}_{I}=0.4$, ${\bar{R}}_{0}=0.1$, and $\bar{J}=0.1$. Figure 13a,b plot the limit cycles of translation and rotation for $\bar{g}=0.8$,$\bar{g}=1.0$, and $\bar{g}=1.2$. Careful calculation shows that for $0.2<\bar{g}<1.8$, the LCE helical spring oscillator is in the self-tension–torsion regime. The energy compensation between energy input and damping dissipation accounts for this result. For a small gravitational acceleration $\bar{g}$, both the light-driven contraction and recovery take place too quickly; the LCE wire only deforms rapidly when passing through the interface between illumination and darkness. For a large gravitational acceleration $\bar{g}$, both the light-driven contraction and recovery are too slow, and the LCE wire is hardly deformed during the oscillation. Therefore, for small or large gravitational accelerations, the net work done by the helical spring through light energy absorption is too small to compensate for the energy dissipated to maintain the oscillation. Figure 13c,d respectively depict the frequencies and amplitudes of translation and rotation under different gravitational accelerations. The amplitudes of self-tension–torsion first increase and then decrease with increasing $\bar{g}$, while their frequencies slightly decrease. These results imply that an appropriate increase in $\bar{g}$ within a certain range can amplify the oscillatory amplitude and improve the conversion efficiency of light energy to mechanical energy in engineering applications.

### 4.8. Influence of the Inertia Moment of Mass Block

**Figure 14:**
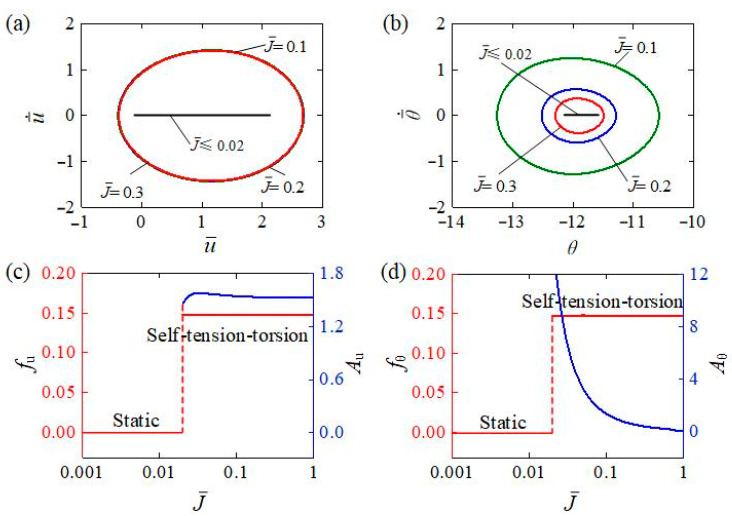
The influence of inertia moment of mass block on the self-tension–torsion for ${\bar{I}}_{0}=0.35$, ${\bar{\beta }}_{1}=0.02$, ${\bar{\beta }}_{2}=0.005$, ${\bar{R}}_{0}=0.1$, ${\bar{E}}_{I}=0.4$, and $\bar{g}=1$. (**a**) Limit cycles for the translation. (**b**) Limit cycles for the rotation. (**c**) Frequency and amplitude of the translation under different inertia moments. (**d**) Frequency and amplitude of the rotation under different inertia moments. As the inertia moment increases, the amplitude of self-tension–torsion for the translation increases slightly at first, and then decreases, while the amplitude for the rotation monotonously decreases and approaches zero.

Figure 14 represents different inertia moments $\bar{J}$ of the mass block affecting the self-tension–torsion for ${\bar{I}}_{0}=0.35$, $C_{0}=0.3$, ${\bar{\beta }}_{1}=0.02$, ${\bar{\beta }}_{2}=0.005$, ${\bar{R}}_{0}=0.1$, ${\bar{E}}_{I}=0.4$, and $\bar{g}=1$. The limit cycles of translation and rotation for $\bar{J}=0.1$, $\bar{J}=0.2$, and $\bar{J}=0.4$ are plotted in Figure 14a,b. There is a critical inertia moment valued at approximately 0.02 to trigger the self-tension–torsion. For a small inertia moment, i.e., $\bar{J}\leq 0.02$, due to the rapid rotation of the mass block, the helical spring may be quickly twisted and untwisted in a short time, and thus both the light-driven contraction and deformation recovery are relatively slow, and the LCE wire barely deforms during the oscillation, inducing too little light energy to be absorbed to compensate for the damping dissipation, and the LCE helical spring oscillator stays in a static regime. The dependences of amplitudes and frequencies on the inertia moment are further shown in Figure 14c,d, respectively. As the inertia moment $\bar{J}$ increases, the amplitude of self-tension–torsion for the translation increases slightly at first and then decreases, while the amplitude for the rotation monotonously decreases and approaches zero. This is also consistent with the physical meaning of inertia moment that the mass block may be difficult to rotate for large $\bar{J}$. Meanwhile, the frequencies for the translation and rotation are independent on the inertia moment. These results imply that appropriately reducing the inertia moment may boost the rotational motion, while the translational motion is virtually unaffected.

## 5. Conclusions

By converting constant external stimuli into mechanical energy, self-oscillation is a superior means of maintaining continuous motion, providing a basis for its widespread use in engineering. Inspired by the self-oscillating system that presents high degrees of freedom and diverse complex oscillatory motions, we created a self-oscillating combined tension–torsion system under steady illumination, among which a mass block and an LCE helical spring made with LCE wire is included. Considering both the well-established helical spring model and the dynamic LCE model, a nonlinear dynamic model of the self-tension–torsion helical spring oscillator under steady illumination was proposed, and the derivation of the corresponding governing equations was carried out.

Upon exposure to steady illumination, the helical spring oscillator was numerically calculated to possess two motion regimes, distinguished as the static regime and the self-tension–torsion regime. The self-tension–torsion of the helical spring oscillator was interpreted as being generated by the light-driven contraction of LCE wire in illumination, and it behaves in a continuous periodic manner due to the mutual balance between the light energy input and the damping dissipation. Meanwhile, the amplitude of self-tension–torsion mainly depends on $C_{0}$, ${\bar{I}}_{0}$, ${\bar{\beta }}_{1}$, ${\bar{\beta }}_{2}$, ${\bar{E}}_{I}$, $\bar{g}$, ${\bar{R}}_{0}$, and $\bar{J}$, while the frequency is mainly determined by ${\bar{E}}_{I}$ and ${\bar{R}}_{0}$.

In conclusion, the proposed self-tension–torsion system shows the unique features of customizable mechanical properties through structural design, small material strain but large structural displacement, and ease of manufacture. We envision that this system may provide novel designs for soft robotics, energy harvesters, active machinery, and so on.

## Figures and Tables

**Table 1:** Material properties and geometric parameters.

Parameter	Definition	Value	Units
$C_{0}$	Contraction coefficient	0~0.5	/
$\tau_{0}$	*trans*-to-*cis* thermal relaxation time	1~100	ms
$I_{0}$	Light intensity	0~20	kW/m^2^
$\eta_{0}$	Light absorption constant	0.0003	m^2^/(s∙W)
$\alpha_{0}$	Helix angle at stress-free	0~0.1	/
$R_{0}$	Coil radius of the LCE helical spring	0~5	mm
$h_{0}$	Height of the LCE helical spring	0~50	mm
$I_{z}$	Cross-sectional inertia moment of LCE wire	(1~5) × 10^−3^	mm^4^
E	Elastic modulus of LCE wire	1~10	MPa
υ	Poisson′s ratio	0.38~0.5	/
m	Mass of mass block	0~100	g
J	Inertia moment of mass block	(0~2.5) × 10^5^	g∙mm^2^
$\beta_{1}$	Translational damping coefficient	0~0.001	mg∙mm/s
$\beta_{2}$	Rotational damping coefficient	0~0.001	mg∙mm^2^/s

**Table 2:** Dimensionless parameters.

Parameter	${\bar{I}}_{0}$	${\bar{\beta }}_{1}$	${\bar{\beta }}_{2}$	${\bar{E}}_{I}$	${\bar{R}}_{0}$	$\bar{J}$	$\bar{g}$
Value	0~0.5	0~0.03	0~0.01	0.1~0.5	0.09~0.11	0~1	1~3

## Data Availability

The data that support the findings of this study are available upon reasonable request from the authors.
